# Screening for Epstein–Barr virus (EBV) infection status in university freshmen: acceptability of a gingival swab method

**DOI:** 10.1017/S0950268819000335

**Published:** 2019-03-08

**Authors:** J. M. Grimm-Geris, S. K. Dunmire, L. M. Duval, E. A. Filtz, H. J. Leuschen, D. O. Schmeling, S. L. Kulasingam, H. H. Balfour

**Affiliations:** 1 Division of Epidemiology and Community Health, University of Minnesota School of Public Health, Minneapolis, MN 55455, USA; 2 Department of Laboratory Medicine and Pathology, University of Minnesota Medical School, Minneapolis, MN 55455, USA; 3 Department of Pediatrics, University of Minnesota Medical School, Minneapolis, MN 55455, USA

**Keywords:** Epstein–Barr virus (EBV), EBV antibodies, EBV infection status, EBV shedding, EBV vaccine, gingival swab, oral wash

## Abstract

Prophylactic vaccines against Epstein–Barr virus (EBV) are under development. EBV-naïve college freshmen are ideal candidates for an efficacy trial, because their incidence of infectious mononucleosis (mono) during freshman year is as high as 20%. To assess perceptions about mono and a mono vaccine, and to learn if EBV immune status could be determined using a gingival swab rather than phlebotomy, we performed a cross-sectional study of 235 healthy students at the beginning of their freshman year. Subjects completed questionnaires and donated oral washes, gingival swabs and venous blood. Overall, 90% of students found the swab easy to use and 80% preferred the swab over venepuncture. Of the 193 students with sufficient samples, 108 (56%) had EBV antibodies in blood *vs.* 87 (45.1%) in the gingival swab. The sensitivity and specificity of the swab compared with blood for detecting EBV antibodies was 75.9% and 94.1%, respectively, with an accuracy of 89.3%. EBV DNA was detected in the oral wash and swab of 39.2% and 30.4% of blood-antibody-positive individuals, respectively. In conclusion, 44% of our freshmen were EBV-naïve and thus vaccine candidates, the gingival swab was an acceptable alternative to phlebotomy for detecting EBV antibody but needs improved sensitivity, and the perceived value of EBV vaccine was high (72% believed they would benefit).

## Introduction

Epstein–Barr virus (EBV) is a ubiquitous human herpesvirus best known for causing infectious mononucleosis (mono), which is noteworthy for its long duration of acute illness that averages 18 days [1]. However, mono is only one of many health problems associated with EBV. This virus causes several forms of cancer, including Burkitt lymphoma, nasopharyngeal carcinoma and Hodgkin lymphoma [2–5], and is implicated in the pathogenesis of autoimmune diseases such as multiple sclerosis [6].

Vaccines designed to prevent or modify primary EBV infection are being developed [7–9], and have potential to reduce the incidence and severity of all EBV-associated diseases. For a vaccine efficacy trial, the subjects should be EBV-naïve and at a relatively high risk of contracting mono. EBV-naïve U.S. college freshmen are ideal candidates for such a trial, because 40%–50% of them are EBV-naïve when they start school and their incidence of mono during freshman year has been reported to be as high as 20% [10, 11].

The usual way to determine EBV infection status (naïve *vs.* previously infected) is to test venous blood obtained by phlebotomy for the presence of EBV-specific antibodies. We reasoned that a gingival swab might be more acceptable to subjects and could enhance screening and enrolment in EBV vaccine trials.

The use of oral fluids as a non-invasive source for detecting antibodies against various pathogens was first established in 1987 [12, 13]. Gingival crevicular fluid (GCF), a component of oral fluids, has since been identified as the primary source of viral antibodies due to its high content of immunoglobulin G (IgG). The method for collecting and testing GCF for IgG antibodies against EBV was first described by Vyse *et al*. in 1997 [14]. Sampling is achieved by rubbing a swab along the gum lines. The assay used for quantification was a ‘G’ antibody capture radioimmunoassay, which limits the usability by researchers due to potential radiation exposure. Despite several modifications, these assays are technically challenging and labour intensive, making them impractical to use in clinical settings.

Thus, the aims of this study were to examine the current prevalence of EBV infection at the beginning of college enrolment, learn about student attitudes concerning mono and a vaccine to prevent it and investigate the feasibility of a gingival swab method for detection of EBV antibodies using a commercially available assay.

## Methods

### Study design

We conducted a cross-sectional study of freshmen students ⩾18 years who were enrolled, questioned and sampled during their first 2 months at the University of Minnesota. Freshmen were selected because they have previously been found to have the lowest prevalence of EBV antibodies compared with sophomores, juniors and seniors [10], which is ideal for assessment of the gingival swab's sensitivity and specificity.

### Recruitment

This study was approved by the University of Minnesota Institutional Review Board and all participants provided written informed consent. Students were recruited from residence halls selected for their proximity to the Clinical Virology Research Laboratory with assistance from the University of Minnesota Housing and Residential Life. Emails were sent to potential participants informing them of the study requirements and inviting them to participate in study sessions in the residence halls, during which research team members obtained consent from eligible participants, and collected the questionnaires and samples.

### Collection of questionnaires and virology samples

Subjects completed a demographic/history questionnaire that included date of birth, sex, race/ethnicity and history of diagnosed mono. Participants were also asked whether or not they thought they had been exposed to EBV, using a five-point Likert scale of certainty.

Participants were given a SalivaBio oral swab (Salimetrics, Carlsbad, CA, USA) (10 × 30 mm) and instructed to swab along their gums for a total of 1 min (15 s per quadrant) under supervision by the study team. Participants also provided a 10 mL sample of venous blood and an oral wash, which consisted of gargling 22 mL of sterile saline (0.9% NaCl) for 30 s and depositing it in a sterile 50 mL tube. After specimen collection, participants were asked to complete an anonymous survey on the acceptability of using the gingival swab and whether they believed they would benefit from an EBV vaccine. Students received a $10 gift card and interpretation of their EBV antibody status for their participation.

### Sample processing

Gingival swabs were centrifuged at 22 000 rpm for 20 min. The fluid retained after processing the swab was considered to be GCF. Volumes less than 100 μL were considered to be insufficient for antibody testing and excluded from analysis. After GCF was collected and stored in a microcentrifuge tube, swabs were washed with 1.0 mL of phosphate buffered saline (PBS) to remove any remaining cells or viral particles. Washed gingival swabs were centrifuged at 22 000 rpm for 10 min to collect diluted GCF (dGCF). Oral wash samples were split into two 10 mL aliquots and centrifuged to collect oral cells and supernatant. The cell pellet was resuspended in PBS, transferred to a 1.5 mL microcentrifuge tube and spun in an Eppendorf microfuge at 14 000 rpm for 1.5 min. Supernatant fluid was then removed using a transfer pipette. Blood samples were centrifuged and 1.0 mL of plasma was saved. All samples were stored at −80 °C until subsequent testing.

### Determination of EBV infection status

EBV infection status is usually assessed by testing for circulating IgG class antibodies against EBV viral capsid antigen (VCA IgG) or IgG class antibodies against EBV nuclear antigen-1 (EBNA-1 IgG). We chose to test for VCA IgG because 5/62 prospectively followed subjects (8%) never developed EBNA IgG antibodies after primary EBV infection, whereas 66/66 developed antibodies against VCA IgG [10].

We used commercial semi-quantitative EIA kits (Diamedix, Miami, FL). We tested plasma instead of serum, after determining the equivalence of those matrices by testing 45 paired, blinded samples. Samples were diluted according to the manufacturer's instructions at a ratio of 1:21 while gingival swab samples were run undiluted. EIA indices, which are referred to hereafter as relative antibody units (RU), were calculated using the formulas in the kit package insert. Data were recorded as positive (EIA index ⩾1.10), negative (EIA index <0.90) or equivocal (EIA index 0.90–1.09). Equivocal samples were repeated and if the samples were still equivocal, those participants were excluded from the final analysis.

### Quantification of EBV by polymerase chain reaction

EBV DNA was extracted from oral cells and dGCF samples, and real-time quantitative polymerase chain reaction (qPCR) was performed as previously detailed [15]. The amplicon is a 71-bp portion of the EBNA-1 gene. The reliable limit of detection is four copies per reaction, or 16 copies/mL. Quantitative EBV data were expressed as copies of EBV DNA/mL of sample. All samples were tested, regardless of antibody status, because viral shedding of EBV can precede the production of antibody in primary infection [11].

### Statistical analysis

Statistical analysis was performed using SAS, version 9.4 (SAS Institute, Cary, NC) or Prism software (Graphpad, La Jolla, CA). Sensitivity, specificity and positive and negative predictive values were calculated using standard methods and exact 95% confidence limits [16]. Agreement between the swab and the blood standard protocols was assessed using Cohen's *κ* coefficient. Receiver operator characteristic (ROC) curves and corresponding area under the curve (AUC) analyses were used to assess the swab's prognostic capabilities. Volumes of gingival swab fluid were grouped into tertiles of ‘low volume’ (0.05–0.1 mL), ‘moderate volume’ (0.2–0.3 mL) and ‘high volume’ (⩾0.4 mL). Logistic regression was performed on variables of interest to assess risk of sampling differences. Adjusted models were assessed for confounding and effect modification.

## Results

### Demographics

Between 29 September and 26 October 2017, a total of 235 University of Minnesota freshman students enrolled in the study; 145 (62%) identified as female. The participants were 88% White, 8% Asian, 2% African American and 2% other racial/ethnic groups.

Since the inclusion criterion was freshmen, there was little variation in age (mean: 18.6 years; range 18–19 years).

### Samples collected and EBV antibody prevalence

**Fig. 1.**
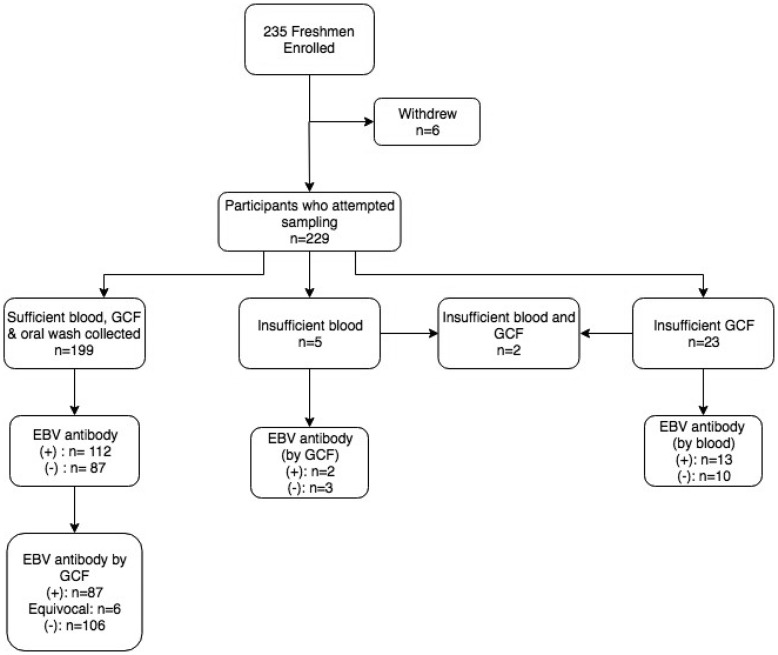
Enrolment summary and corresponding EBV antibody prevalence. Antibody prevalence was determined using the gold standard method of blood plasma, unless noted. (+)* = EBV VCA IgG antibody-positive; (−)* = EBV VCA IgG antibody negative; *cut-off values previously described.

Figure 1 displays the enrolment summary and EBV antibody prevalence. Six of the 235 students enrolled but withdrew before sample collection. Thirty (13%) of the 229 students who attempted sample collection did not have a sufficient volume of gingival swab fluid or blood for antibody testing. All 229 students completed an oral wash. Of the 199 students who successfully provided all three samples, six subjects had equivocal gingival swab antibody results, leaving 193 subjects for comparison of antibody status.

Overall, 112 (56.3%) of students had EBV VCA IgG antibodies in their plasma. The prevalence of EBV antibodies did not differ by individuals who were not included in the final analysis due to inadequate sample collection (*P* = 0.4). There was no significant difference in the proportion of females who were EBV antibody-positive compared with males (*P* = 1.0).

### Comparison of EBV antibodies in blood and gingival swabs

Table 1 presents the results of the gingival swab compared with plasma for detecting EBV antibodies. Of the 193 students with sufficient volumes of plasma and gingival swabs, 108 (56.0%) were positive for EBV VCA IgG antibodies in plasma and 87 (45.1%) in GCF (*P* = 0.03). The mean RU of VCA IgG among EBV antibody-positive individuals was higher in plasma samples than gingival swabs (*P* = 0.002), 3.94 and 2.57 RU, respectively.

**Table 1.** EBV EIA antibody results of GCF compared with blood plasma

Measurement	Blood plasma	GCF
Proportion positive (%)	108/193 (56.0%)^a^	87/193 (45.1%)^a^
Mean RU (s.d.)^b^	3.94 (1.39)^c^	2.57 (1.34)^c^
Test measurement (95% CI) of GCF to plasma		
Sensitivity	Reference	75.9% (67.9%, 84.0%)
Specificity		94.1% (89.1%, 99.1%)
Positive predictive value		94.3% (89.4%, 99.1%)
Negative predictive value		75.5% (67.3%, 83.7%)
Positive likelihood ratio		12.9 (5.5, 30.4)
Negative likelihood ratio		0.3 (0.2, 0.4)
Accuracy		89.3% (78.0%, 88.8%)
*κ*		0.68 (0.58, 0.78)

a *χ*^2^ test = 4.57, *P* = 0.03.

b Of positive samples.

c Paired *t*-test between GCF and blood plasma, *P* = 0.002.

**Fig. 2.**
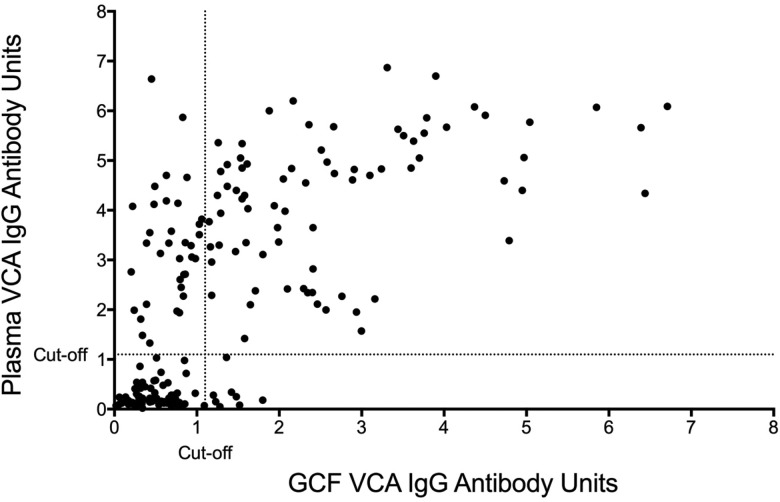
GCF *vs.* blood plasma EBV VCA IgG EIA antibody units. Cut-off by the manufacturer's instructions.

**Fig. 3.**
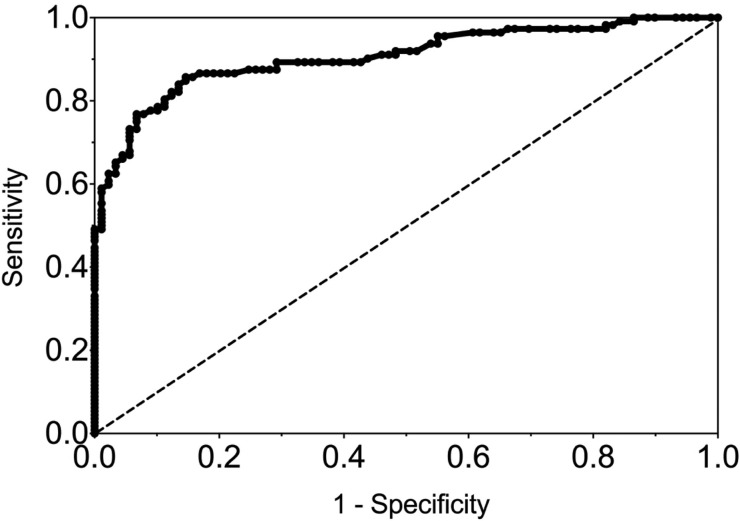
ROC curve of GCF EBV VCA IgG antibody. AUC = 0.91 (0.86, 0.95), *P* < 0.0001.

The sensitivity and specificity of gingival swabs compared with plasma for detecting EBV VCA IgG antibodies was 75.9% [95% confidence interval (CI) 67.9%, 84.0%] and 94.1% (95% CI 89.1%, 99.1%), respectively. This provided an overall accuracy of 89.3% (95% CI 78.0%, 88.8%). Figure 2 displays the paired gingival swabs and plasma EBV VCA IgG antibody unit results. As indicated by the specificity, there were very few false positive samples using the cut-off determined by the kit manufacturer. To assess the appropriateness of the cut-off used, we created a ROC curve of the test's sensitivity *vs.* 1-specificity (Fig. 3). The AUC was 0.91 (95% CI 0.86, 0.95) (*P* < 0.0001).

### Prevalence of EBV DNA in the gingival swab and oral wash

Considering all participants, regardless of EBV antibody status, EBV DNA was present in the oral wash of 48 students (48/199; 24.1%) whose samples had sufficient volume (Table 2). The prevalence of EBV DNA in dGCF was lower (17.6%) as compared with oral cells, but this was not statistically significant (*P* = 0.1).

**Table 2.** EBV DNA recovered from dGCF compared with oral wash

Measurement	Oral cells	dGCF
Proportion positive (%)	48/199 (24.1%)^a^	35/199 (17.6%)^a^
Mean log_10_ copies/mL EBV (range)^b^	3.99 (2.42–6.85)^c^	3.56 (2.33–5.82)^c^
Test measurement (95% CI) of GCF to gold standard		
Sensitivity	Reference	54.2% (39.2%, 68.6%)
Specificity		94.0% (89.0%, 97.2%)
Positive predictive value		74.3% (59.3%, 85.1%)
Negative predictive value		86.4% (78.6%, 89.2%)
Positive likelihood ratio		9.1 (4.6, 18.0)
Negative likelihood ratio		0.5 (0.4, 0.7)
Accuracy		84.4% (78.6%, 89.2%)
*κ*		0.53 (0.39, 0.67)

a *χ*^2^ statistic = 2.57, *P* = 0.1.

b Of antibody-positive samples.

c Paired *t*-test between GCF and oral cell samples, *P* = 0.005.

EBV DNA was present in the oral wash and GCF of 44 and 34 antibody-positive students, respectively (44/112; 39.2%) (34/112; 30.4%) (*P* = 0.2). There was a significant difference in the mean log_10_ copies/mL of EBV in oral cells (3.99 log_10_ copies/mL) compared with dGCF (3.56 log_10_ copies/mL) (*P* = 0.005). The sensitivity and specificity of dGCF compared with oral wash specimens was 54.2% (95% CI 39.2%, 68.6%) and 94.0% (95% CI 89.0%, 97.2%), respectively. The overall accuracy was 84.4% (95% CI 78.6%, 89.2%).

### Effect of gingival swab volume on test measurements

The mean volume obtained from gingival swabs was 0.2 mL (range: 0.0–0.6 mL). Table 3 shows the effect of gingival swab volume on test measurements. There was a significant difference in the proportion of men who had high volumes compared with women: men had 1.93 times higher odds of being in a higher tertile of gingival swab volume compared with women (*P* = 0.02). There was no difference in the mean EBV antibody units or EBV copies/mL among antibody-positive samples by gingival swab volume.

**Table 3.** Effect of GCF volume on test measurements

Measurement	Volume of dGCF			*P* value^a^
	0.05–0.1 mL	0.2–0.3 mL	⩾0.4 mL	
Proportion of samples (%)	86/199 (43.2%)	74/199 (37.2%)	39/199 (19.6%)	0.0001
Proportion female (%)	57/86 (66.3%)	43/74 (58.1%)	17/39 (43.6%)	0.05
Mean RU in GCF (s.d.)^b^	2.2 (1.3)	2.2 (1.5)	1.7 (1.6)	0.3
Mean log_10_ copies/mL EBV in dGCF (s.d.)^b^	1.0 (1.7)	1.0 (1.7)	1.3 (1.8)	0.9

a *χ*^2^ or one-way analysis of variance *F*-test.

b Of antibody-positive samples.

**Fig. 4.**
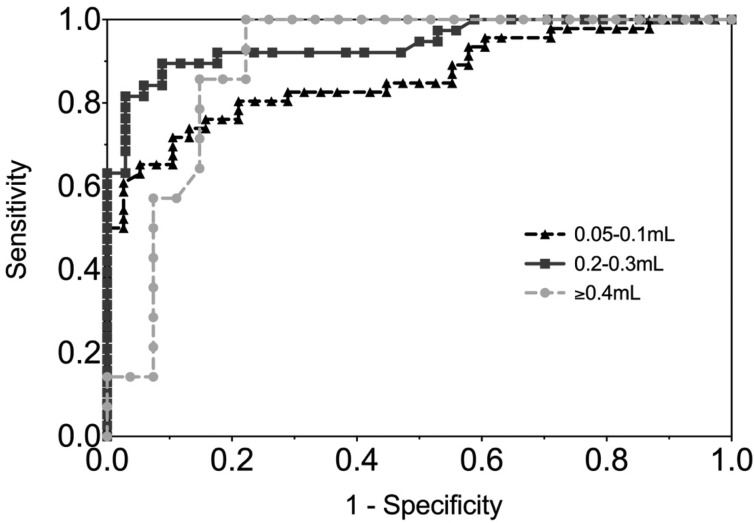
ROC curve of EBV VCA IgG antibody in GCF by volume of GCF collected. 0.05–0.1 mL AUC = 0.86 (0.78, 0.94), 0.2–0.3 mL AUC = 0.94 (0.89, 0.99), ⩾0.4 mL AUC = 0.90 (0.80, 0.99).

Figure 4 shows the ROC curve of EBV VCA IgG antibody in the gingival swab by volume collected. The moderate volume group had the highest AUC of 0.94 (*P* < 0.0001), indicating excellent diagnostic accuracy.

### Attitudes about the gingival swab and value of an EBV vaccine

**Fig. 5.**
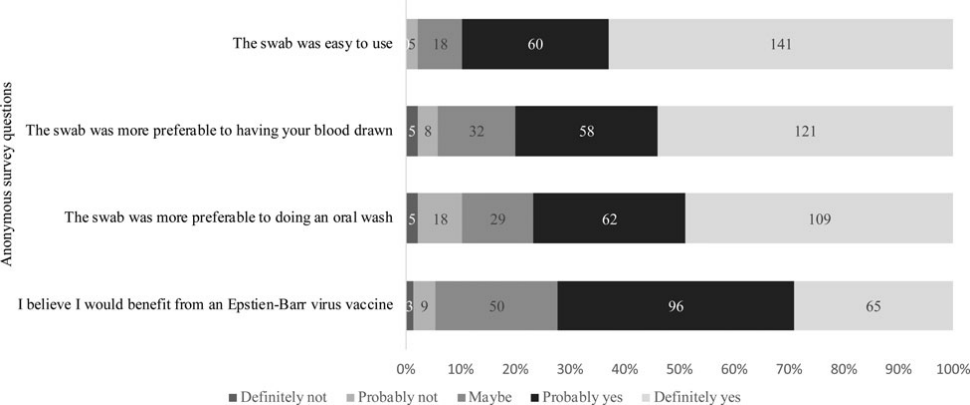
Attitudes regarding the gingival swab and the value of an EBV vaccine. Students were asked to answer based on their agreement to each of the questions stated above.

Of the 229 students who attempted sample collection, 224 completed the anonymous questionnaire regarding their attitudes on using the swab and the value of an EBV vaccine. As shown in Figure 5, 201 (90%) students agreed with the statement that the swab was easy to use. When compared with having their blood drawn or doing an oral wash, 94.2% and 89.7% found the swab more preferable, respectively. Students were also asked about their awareness of EBV and their opinions about an EBV vaccine. Whereas 37% of students reported not knowing about EBV, 72% of students believed they would benefit from an EBV vaccine, regardless of their immune status.

As part of the demographic survey, 13 students reported a laboratory confirmed diagnosis of mono and, indeed, all 13 were EBV antibody-positive. Excluding these participants, 41.4% of students guessed they were either probably or definitely EBV antibody-positive, 32.6% marked ‘unsure’, and 26% said they were probably or definitely EBV negative. Interestingly, we found that individuals who were antibody-positive had 80% lower odds of correctly guessing their antibody status than individuals who were EBV naïve, after controlling for a past diagnosis of mono (*P* < 0.0001). This finding indicates that freshmen tend to underestimate their risk of being EBV carriers.

## Discussion

The aim of this project was to determine if we could screen for EBV antibodies using an oral sample, which would be relatively non-invasive and potentially more acceptable than phlebotomy. We investigated swabbing the gums to collect GCF, which is a transudate of blood from gum capillaries.

The gingival swab had fair sensitivity and specificity (75.9% and 94.1%, respectively) compared with plasma, using the cut-off recommended by the EIA manufacturer, and moderate agreement by the *κ* coefficient value (0.68) [17]. However, if the cut-off was lowered to 0.8, for example, to reduce the number of samples that were classified as false negatives, the sensitivity increased to 82.1% (95% CI 75.1%, 89.2%) while lowering the specificity to 86.2% (95% CI 79.0%, 93.5%). This cut-off is supported by our ROC curve, which showed that a sensitivity and specificity of approximately 80% is the point of maximised performance without compromising specificity.

The sensitivity and specificity may also be improved if differences in GCF volume are taken into account. We found that the ideal volume to maximise accuracy measurements was 0.2–0.3 mL. While on average most samples were in this range, sample volumes ranged from 0.05 to 0.6 mL. Large volumes appeared to be too dilute to obtain a sufficient concentration of IgG antibody, while small volumes did not evenly coat the wells of the EIA plate.

There were differences between men and women in regard to the volumes of GCF collected, which in turn corresponded to differences in the number of false negative samples. Women were more likely than men to have low volume samples. A possible reason for this was that women were more hesitant than men to properly swab their gums.

EBV DNA was detected in 30.4% of antibody-positive individuals in the swab compared with 39.2% in the oral wash. The swab had low sensitivity (54.2%) when compared with the oral wash, but good specificity (94.0%). An explanation for this phenomenon may be the differences in the location and tissue types from where the samples are being collected.

Based on the survey data collected, 90% of students agreed that the swab was easy to use for sample collection. One reason the swab may have been difficult for some students was because participants had to directly hold on to the swab while brushing their gums. However, in general, students agreed that the gingival swab was preferable to phlebotomy and an oral wash, (94.2% and 89.7% of students found it more preferable, respectively). To estimate the receptiveness to an EBV vaccine in this population, we asked students whether they believed they would benefit from a vaccine, regardless of their immune status; 72.2% said they believed they would benefit and 22.4% said they might benefit.

This study has several limitations. The volume of GCF collected from the swab was not uniform across samples, a finding similar to other studies [18, 19]. Nokes *et al*. found variation in the concentration of IgG in GCF by various swab methods [20]. There was also no assurance with this method that a sufficient volume had been obtained or that it was the appropriate type of fluid [21].

There were also limitations with its ease of use. As previously mentioned, due to the swab's design, participants had to directly hold onto the swab while brushing it against their gums which caused some participants to drop the swab mid-collection. Also, antibody index values of GCF were not directly proportional to index values of plasma. Therefore, the swab may best be used for the qualitative assessment of EBV antibody status.

Despite these limitations, the swab was an easy, non-invasive and relatively inexpensive method for detecting EBV antibodies compared with blood. The cost of the swab was $1.72 *vs.* $2.50 for blood drawing supplies plus the cost of a trained phlebotomist. The swab can also be used as a two-in-one method, because it was able to detect virus in the oral cavity.

In summary, a gingival swab could provide a highly acceptable and relatively non-invasive alternative to phlebotomy for screening for EBV, particularly for future EBV vaccine trials. We showed that university freshmen are indeed an ideal adult population to be vaccinated since 44% were EBV naïve, and their interest in an EBV vaccine was high. In terms of future directions, we would like to investigate other swab devices in order to maximise the ease of use and potentially obtain better sensitivity and specificity of EBV VCA IgG antibody detection.
